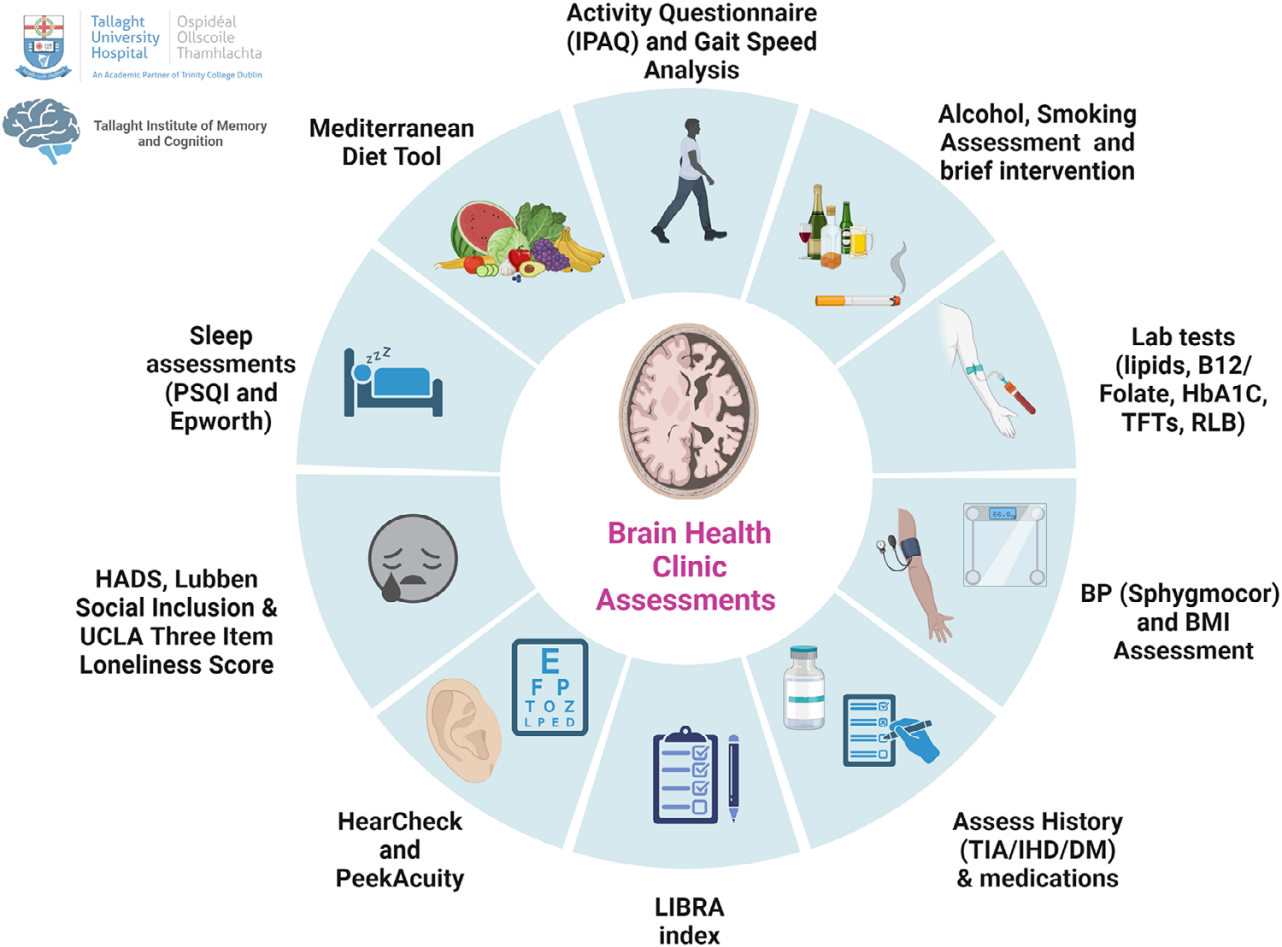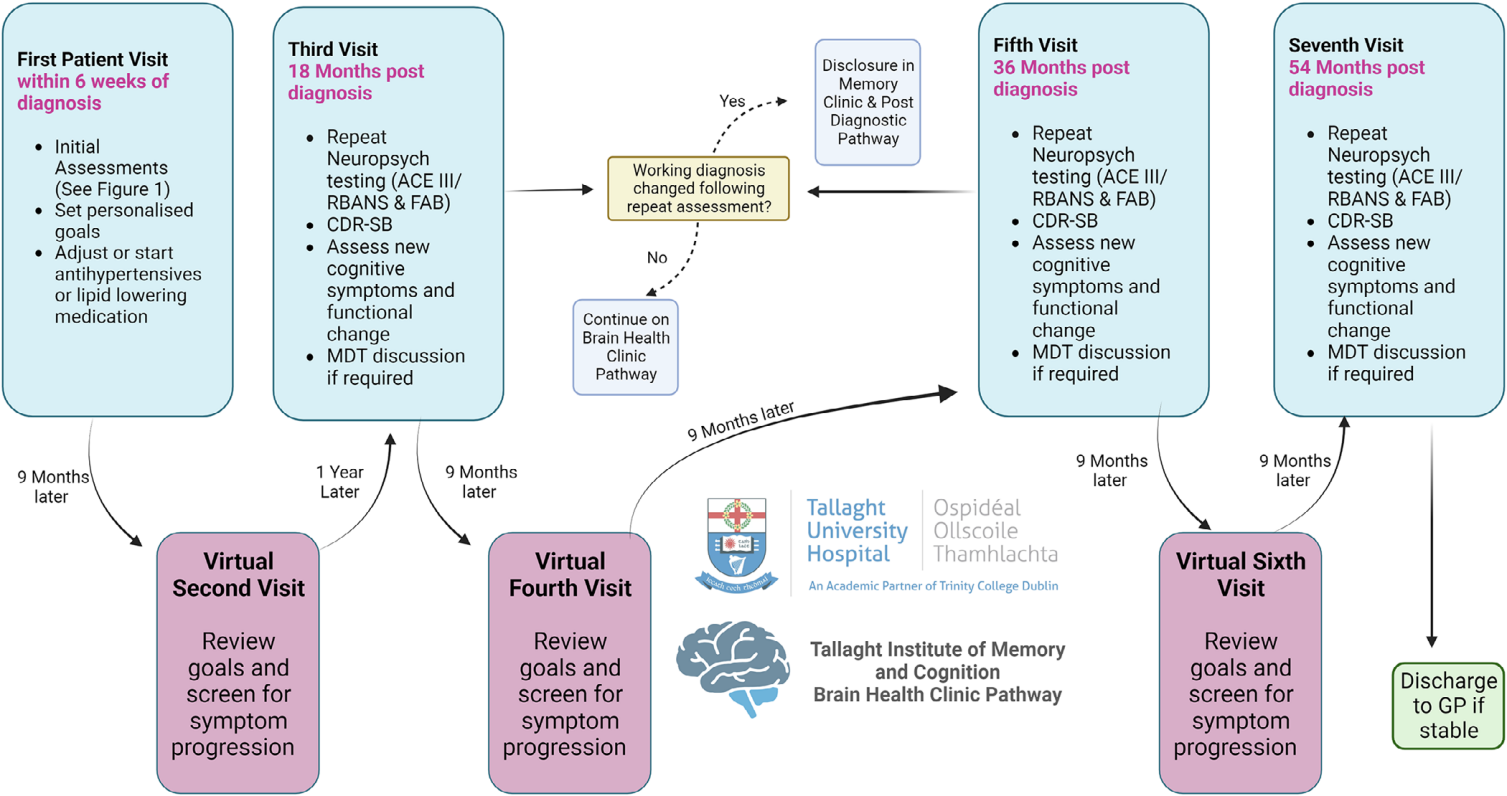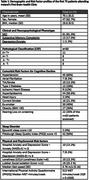# Brain Health Interventions in Clinical Practice: A Framework for Implementation and Provisional Results from Ireland’s first Brain Health Clinic

**DOI:** 10.1002/alz.087890

**Published:** 2025-01-09

**Authors:** Eimear Connolly, Helena Dolphin, Graham Knight, Aoife Fallon, Sean P Kennelly

**Affiliations:** ^1^ Tallaght University Hospital Institute of Memory and Cognition, Dublin Ireland

## Abstract

**Background:**

At a population level, it is acknowledged that around 40% of all causes of dementia are attributable to twelve potentially modifiable risk factors (MRFs) identified by The Lancet Commission on Dementia Prevention. Preventative measures that target these MRFs should be integrated into clinical practice, alongside biomarker‐supported diagnosis of Mild Cognitive Impairment (MCI) or Dementia. However, most memory services do not routinely provide a preventative Brain Health Clinic (BHC).

**Methods:**

Tallaght University Hospital Institute of Memory and Cognition (TIMC) is a Regional Specialist Memory Clinic (RSMC) in an Irish tertiary hospital which provides patients with biomarker‐supported multidisciplinary consensus diagnosis. It has established Ireland’s first BHC to identify and address MRFs for dementia. Here we outline a framework for implementation of a BHC integrated within a clinical memory service. The assessment tools used to capture specific MRFs will be detailed. Data has been collected from the first 75 patients attending the service, and their demographic details, consensus diagnosis and MRF profiles will be described.

**Results:**

The assessments carried out in our BHC are outlined in Figure 1. The BHC Pathway is shown in Figure 2. Patients are followed longitudinally with repeat cognitive testing at 18, 36 and 54 months. The demographic data for the first 75 patients attending this service, their consensus diagnosis and MRF profiles are outlined in Table 1. The most common diagnosis was MCI (n = 61) of which 48 had an amnestic phenotype. Cerebrospinal Fluid (CSF) biomarkers for Alzheimer’s Disease (AD) pathology were tested in 32 patients (52.45% of MCI patients). Hypertension was the most evident vascular risk factor, found in 60% of participants. Over half of patients had self‐reported poor sleep using the Pittsburgh Sleep Quality Index (PSQI). Hearing was assessed in 63 patients (84%), with 35% (n = 22) of tested patients requiring referral for formal audiology testing, highlighting the importance of screening for this risk factor.

**Conclusions:**

TIMC have implemented a BHC and embedded it within a RSMC model. This enables patients to engage in targeted, personalised MRF assessment and modification. The risk factor profile of patients attending our BHC provides a valuable insight into MRF prevalence in patients attending memory clinics.